# Prevalence of IgG antibodies against SARS-CoV-2 among healthcare workers in a tertiary pediatric hospital in Poland

**DOI:** 10.1371/journal.pone.0249550

**Published:** 2021-04-01

**Authors:** Beata Kasztelewicz, Katarzyna Janiszewska, Julia Burzyńska, Emilia Szydłowska, Marek Migdał, Katarzyna Dzierżanowska-Fangrat

**Affiliations:** 1 Department of Clinical Microbiology and Immunology, The Children’s Memorial Health Institute, Warsaw, Poland; 2 Department of Anaesthesiology and Intensive Care, The Children’s Memorial Health Institute, Warsaw, Poland; Universidad Nacional de la Plata, ARGENTINA

## Abstract

Data on the prevalence of the SARS-CoV-2 antibody in healthcare workers (HCWs) is scarce, especially in pediatric settings. The purpose of this study was to evaluate SARS-CoV-2 IgG-positivity among HCWs of a tertiary pediatric hospital. In addition, follow-up of the serological response in the subgroup of seropositive HCWs was analysed, to gain some insight on the persistence of IgG antibodies to SARS-CoV-2. We performed a retrospective analysis of voluntary SARS-CoV-2 IgG testing, which was made available free of charge to HCWs of the Children’s Memorial Health Institute in Warsaw (Poland). Plasma samples were collected between July 1 and August 9, 2020, and tested using the Abbott SARS-CoV-2 IgG assay. Of 2,282 eligible participants, 1,879 (82.3%) HCWs volunteered to undergo testing. Sixteen HCWs tested positive for SARS-CoV-2 IgG, corresponding to a seroprevalence of 0.85%. Among seropositive HCWs, three HCWs had confirmed COVID-19. Nine (56.3%) of the seropositive HCWs reported neither symptoms nor unprotected contact with confirmed SARS-CoV-2 cases in the previous months. A decline in the IgG index was observed at a median time of 86.5 days (range:84‒128 days) after symptom onset or RT-PCR testing. Further studies are necessary to elucidate the duration of persistence of anti-SARS-CoV-2 antibodies, as well as the correlation between seropositivity and protective immunity against reinfection. Regardless of the persistence of antibodies and their protective properties, such low prevalence indicates that this population is vulnerable to a second wave of the COVID-19 pandemic.

## Introduction

Severe acute respiratory syndrome coronavirus 2 (SARS-CoV-2), causing the coronavirus disease 2019 (COVID-19) which emerged in December 2019, has evolved into a global pandemic [1]. In Poland, the first imported COVID-19 case was reported on March 3, 2020, and three weeks later a nationwide lockdown was commenced [2]. Up to August 31, there were 66,870 confirmed cases, with 2,033 COVID-19 related deaths [3].

In the Masovian district (one of the three most affected regions in Poland), the first cases were recorded on March 13 and by the end of August 2020 there were 9,370 cases and 411 deaths [4].

Although real-time RT-PCR is considered the gold standard for the diagnosis of acute SARS-CoV-2 infection, this test is limited by the transient nature of RNA. In addition, the sensitivity of RT-PCR methods is imperfect [5], which may lead to the underdiagnosing of SARS-CoV-2 infections, especially in subclinical or asymptomatic cases. By identifying individuals who have developed antibodies to the virus (including those who may be asymptomatic or have recovered), serology can give greater insight into the prevalence of SARS-CoV-2. However, concerns have arisen on the persistence of IgG antibodies to SARS-CoV-2 after recovery [6, 7].

Two groups of infected individuals pose the highest risk for SARS-CoV-2 transmission in a hospital setting: infected patients before diagnosis and SARS-CoV-2-positive health care workers (HCWs). As children and adolescents make up less than five percent of all positive cases in Europe [8], the majority of SARS-CoV-2 infections among HCWs in pediatric hospitals is probably associated with transmission in the community or from infected co-workers.

Data on SARS-CoV-2 prevalence among HCWs in pediatric hospital settings is scarce [9, 10].

Knowing the prevalence of SARS-CoV-2 infection among HCWs is vital for an informed pandemic response. The aim of this study was to evaluate SARS-CoV-2 IgG positivity among HCWs of a tertiary pediatric hospital in Warsaw (Masovian district), Poland. In addition, we have performed follow-up of the serological response to SARS-CoV-2 in the subgroup of seropositive HCWs to gain some insight on the persistence of specific antibodies.

## Materials and methods

The study was a retrospective analysis of data from voluntary SARS-CoV-2 IgG testing performed among HCWs at The Children’s Memorial Health Institute (CMHI) in Warsaw, Poland. Testing was made available for all HCWs (including physicians, nurses, and other workers with direct patient contact, i.e. physical therapists, as well as workers without direct patient contact, i.e. laboratory workers, pharmacists, administrative staff, maintenance staff, etc.). All participants were asymptomatic at the time of serology testing. In particular, the study included participants who were previously symptomatic but had no symptoms for at least 14 days. Plasma (EDTA) samples were collected between July 1 and August 9, 2020, (corresponding to 97‒135 days after the nation-wide lockdown was commenced). Plasma samples were tested on the Abbott Alinity i instrument using the Abbott SARS-CoV-2 IgG assay (Abbott Laboratories, Lake Bluff, IL, USA) following the manufacturer’s instructions. The assay is a chemiluminescent microparticle immunoassay (CMIA) for the qualitative detection of IgG antibodies to the nucleocapsid (N) protein of SARS-CoV-2. Briefly, in this assay, IgG antibodies to SARS-CoV-2 present in the sample bind to SARS-CoV-2 antigen-coated microparticles. After applying an anti-human IgG acridinium-labeled conjugate, the resulting chemiluminescent reaction is measured as a relative light unit (RLU). There is a direct relationship between the amount of IgG antibodies to SARS-CoV-2 in the sample and the RLU detected by the system optics. This relationship is reflected in the calculated index (a signal/cut-off; S/CO, ratio). The manufacturer’s index value of ≥1.40 was interpreted as positive. The assay has been shown to have a 99.9% specificity and 100% sensitivity for samples taken greater than 17 days post symptom onset [11].

In addition, we report retrospective data in a subgroup of HCWs with multiple SARS-CoV-2 IgG test results available. The follow-up serology tests were performed in the same way as the initial test described above. The HCWs with follow-up had initially tested seropositive or were initially seronegative but subsequently contracted SARS-CoV-2 and were identified by the Infection Control Department as part of an epidemiological investigation. Demographic data (age, gender) and the results of SARS-CoV-2 RNA testing (if performed at the CMHI) were collected for all participants, from laboratory records. Data on profession and the necessity of quarantine or isolation (date and duration of quarantine or isolation) were collected from the human resource’s database. In addition, in the case of seropositive infected individuals, data from the Infection Control Department records were retrieved regarding contacts with a confirmed or a suspected COVID-19 case, positive test results in the past (if performed outside the CMHI), the necessity of inpatient treatment, and symptoms experienced over the previous months. The data was de-identified by the head of the Infection Control Department prior to analysis.

All data were analyzed anonymously in October, 2020.

### Statistical analysis

Statistical analysis was carried out using the Statistica data analysis software system (TIBCO Software Inc.), version 13. Continuous variables were presented as median and interquartile range (IQR). Categorical variables were summarized using percentages and counts. Seroprevalence of SARS-CoV-2 IgG was calculated as a proportion with 95% confidence intervals (CI). The association between variables was tested with the Chi-squared or Fisher’s exact test (for categorical variables) and the Mann Whitney U test (for continuous variables). Univariable and multivariable logistic regression analysis were run to evaluate factors associated with the seroprevalence of SARS-CoV-2 IgG. For the variables to be included in the multiple logistic model, a stepwise selection was used, starting with the full model, and using a p-value of 0.1 for the removal and 0.05 for the addition of variables.

We introduced the subgroup analysis due to the fact that HCWs in clinical settings are at a higher risk of becoming seropositive. We assumed a different risk for clinical and non-clinical settings (or for those HCWs with direct or indirect patient contact) based on the fact that a proportion of nurses and physicians and other HCWs with direct patient contact worked in multiple healthcare settings both in CMHI outpatient clinics, where patients were not screened for SARS-CoV-2, as well as outside CMHI, where they might be exposed to infection (especially at the beginning of the pandemic). In addition, we assumed that clinical staff was also more exposed than administrative staff due to the higher number of daily contacts not only with patients but also with caregivers and co-workers. Although we introduced screening of patients and their caregivers at hospital entry, the screening of HCWs was limited to symptomatic individuals or was performed as part of contact tracing, therefore we could not completely rule out the possibility of asymptomatic infections among HCWs. On the other hand, the administrative staff in our hospital was allowed to work remotely whenever possible and the number of contacts with other HCWs was substantially lowered as documents management in CMHI was limited to electronic flow only.

### Ethical consideration

This study reports the results of voluntary serology testing which was offered as a free service to healthcare workers and not as part of a research protocol. The study has been reviewed and approved by the Institutional Review Board of the Children’s Memorial Health Institute in Warsaw (Ref. no. 10/P-IN/20), and granted a waiver of consent since the data were retrospective and anonymized before access and analysis. Data were accessed between September and October, 2020. Individuals who tested positive were contacted by the Head of the Infection Control Department as a part of an epidemiological investigation and thus additional data were available. The individuals referred to in this manuscript have given their written informed consent (as outlined in the PLOS consent form) to publish their case details.

## Results

### Study setting

The CMHI in Warsaw (Masovian district) is the largest tertiary pediatric hospital and research institute in Poland. With over 590 beds and 2,282 employees, the CMHI performs over 249,000 services (both inpatient and outpatient) per year.

**Fig 1:**
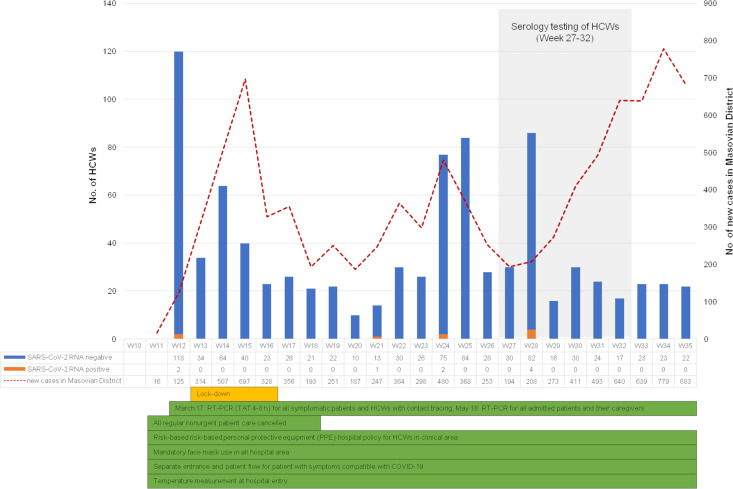
A weekly timeline of the COVID-19 epidemic. The epidemic curve is shown as the number of HCWs tested each week for SARS-CoV-2 RNA by RT-PCR at the CMHI together with the number of new SARS-CoV-2 cases recorded in the Masovian district. The first SARS-CoV-2-positive case among HCWs of the CMHI was detected on March 17, 2020. Voluntary serological testing for HCWs of the CMHI was conducted from July 1 to August 9, 2020 (corresponding to week 27 and 32). Details of infection prevention and control measures implemented at the CMHI together with the nationwide lockdown, are given below the graph.

At the time of the serology testing, we had no cases of SARS-CoV-2 infection among the inpatients. Up to July1, 2020, the first day of the serology testing, we had had five confirmed SARS-CoV-2 infections among HCWs (all contracted outside the hospital setting, one confirmed test outside the CMHI) and there were no additional cases until July 6, 2020. From July 6 up to August 9, 2020 (i.e. the end of the serology testing), an additional four linked cases creating a cluster among the laboratory staff, were confirmed by RT-PCR (Fig 1).

### Baseline characteristics

Of 2,282 eligible participants, 1,879 HCWs volunteered to undergo testing, yielding a participation rate of 82.3%. Median (IQR) age was 48 (38‒56) years and 85.8% were female. Approximately one third (639 out of 1,879, 34%) were nurses, and 19.7% were physicians.

The majority (70.9%) of HCWs worked in the clinical area. Twenty-two point two percent (417 out of 1,879) had been tested for SARS-CoV-2 RNA by RT-PCR as part of the implemented infection control measurements, i.e. contact tracing, for previously quarantined or isolated HCWs before returning to work or for newly employed staff. The median time between RT-PCR and serology testing was 77 days (IQR: 39‒122 days). SARS-CoV-2 RNA was detected in 4 out of 417 HCWs, including 3 HCWs with positive serology (details given below). The remaining one, with a positive SARS-CoV-2 RNA test, was seronegative. This was the HCW whose plasma was collected 129 days post RT-PCR testing.

Fifty-six HCWs had been quarantined or isolated. The median time since the start of quarantine or isolation to serology testing was 97.5 days (IQR: 42.5‒126 days).

See Table 1.

**Table 1:** Baseline characteristics of 1879 HCWs.

Characteristics	Total
Gender:	
male	266 (14.2%)
female	1,613 (85.8%)
Age, median (IQR) years	48 (38‒56)
Professional category:^a^	
nurse	639 (34.0%)
physician	371 (19.7%)
other with direct patient contact	226 (12.0%)
other without direct patient contact	643 (34.2%)
Healthcare department:^b^	
clinical	1,332 (70.9%)
non-clinical	547 (29.1%)
Previously tested for SARS-CoV-2 RNA	
yes^c^	417 (22.2%)
no	1,462 (77.8%)
Quarantined or isolated:	
yes^d^	56 (3.0%)
no	1,823 (97.0%)

^a^*other with direct patient contact*: patient care technician (67), physical therapist (43), radiation therapist (37), psychologist (21), medical assistant (11), audiologist (10), pedagogue (9), speech pathologist (8), clinic engineer (6), medical technician (6), dental assistant (4), anthropologist (4); *other without direct patient contact*: office worker (228), secretary (136), laboratory worker (110), kitchen worker (34), pharmacist (32), dietician (27), medical sterilization technician (19), driver (17), labourer (15), store person (8), IT worker (7), public health worker (3), manager (2), security officer (2), chaplain (2), parking attendant (1).

^b^*clinical departments* include: medical (765), surgical (222), auxiliary medical (123), ambulatory (116), intensive care (106); *non-clinical departments* include: administration (284), laboratory (113), maintenance (116), pharmacy (34).

^c^ median period between SARS-CoV-2 RNA and serology testing was 77 days (range 1–136 days; IQR 39‒122 days); 4 out of 417 (0.96%) HCWs had positive test results.

^d^ median period between the start of quarantine/isolation and serology testing was 97.5 days (range 10‒133 days; IQR 42.5‒126 days).

### Seroprevalence among HCWs

Sixteen healthcare workers tested positive for SARS-CoV-2 IgG, corresponding to a seroprevalence of 0.85%. The median index was 2.69 S/CO (range: 1.41‒7.59 S/CO).

Of the 16 seropositive HCWs, only 3 (18.75%) tested positive for SARS-CoV-2 RNA (within the previous 53‒106 days). Five seropositive HCWs were RT-PCR negative within the prior 6‒127 days (only one of them had been tested less than 14 days prior to serology testing). The remaining eight seropositive HCWs had not been tested by RT-PCR (all of them were negative, when tested 1–11 days after IgG testing).

Among seropositive HCWs, six (37.5%) presented symptoms compatible with COVID-19, one had a household contact with a suspected COVID-19 case, whereas 9 (56.3%) reported neither symptoms compatible with COVID-19 in the previous months nor unprotected close contacts with confirmed or suspected COVID-19 cases. See Table 2.

**Table 2:** Characteristics of the seropositive HCWs.

No.	Age (years)	Gender	Professional category	SARS-CoV-2 IgG (S/CO)	SARS-CoV-2 RNA by RT-PCR	Time since RT-PCR testing and serology testing	Symptoms compatible with COVID-19	Possible route of SARS-CoV-2 transmission
1	48	f	physician	3.07	negative	2 days*	no	unknown
2	44	f	other without direct patient contact	1.85	negative	2 days*	no	unknown
3	56	f	nurse	1.52	negative	11 days*	no	unknown
4	61	f	other without direct patient contact	1.41	negative	6 days	no	unknown
5	61	f	physician	5.00	positive	43 days	yes	household contact with a confirmed case
6	49	f	other with direct patient contact	1.66	negative	3 days*	no	unknown
7	51	f	nurse	6.92	negative	1 day*	no	household contact with a suspected case
8	44	f	physician	2.04	(multiple) negative	14, 34, 42, 111 days	yes	unknown
9	39	f	physician	2.42	(multiple) negative	62, 127 days	no	unknown
10	53	m	physician	2.86	positive	106 days	yes	unknown
11	54	f	nurse	4.32	negative	82 days	no	unknown
12	40	f	physician	2.51	negative	within 24 hours*	no	unknown
13	50	f	nurse	3.76	positive	53 days	yes	unknown
14	59	f	other without direct patient contact	7.59	negative	14 days	yes	unknown
15	65	m	physician	2.21	negative	2 days*	no	unknown
16	49	f	other without direct patient contact	7.32	negative	within 24 hours	yes	unknown

* Indicates HCWs who were tested by RT-PCR after serology testing results were obtained.

### Factors associated with SARS-CoV-2 IgG positivity

The odds of being seropositive were higher in HCWs who had been previously tested by RT-PCR regardless of the test results (adjusted OR = 3.82, 95% CI: 1.42‒10.29; p = 0.008) (Table 3). Age, gender, professional category, or working in a clinical area did not show any statistically significant association with positivity for SARS-CoV-2 IgG (Table 3).

**Table 3:** Analysis of factors associated with SARS-CoV-2 IgG positivity.

	SARS-CoV-2 IgG		p-value^a^	Univariate		Multivariate	
Characteristics	negative	positive		OR (95% CI)	p-value	OR (95% CI)	p-value^c^
Gender:			0.849		0.849		
male	264 (14.2%)	2 (12.5%)		1			
female	1599 (85.8%)	14 (87.5%)		1.16 (0.26‒5.11)			
Age, median, IQR; years^b^	48 (38‒56)	50.5 (46‒57.5)	0.141	1.03 (0.99‒1.08)	0.131	1.04 (0.99‒1.09)	0.093
Professional category:			0.114				
other without direct patient contact	639 (34%)	4 (25%)		1			
nurse	635 (34%)	4 (25%)		1.01 (0.25‒4.04)	0.992		
physician	364 (20%)	7 (44%)		3.07 (0.89‒10.56)	0.075		
other with direct patient contact	225 (12%)	1 (6%)		0.71 (0.08‒6.39)	0.760		
Healthcare department:			0.716		0.717		
non-clinical	543 (29%)	4 (25%)		1			
clinical	1320 (71%)	12 (75%)		1.23 (0.40‒3.84)			
Previously tested for SARS-CoV-2 RNA			0.007		0.012		0.008
no	1454 (78.05%)	8 (50%)		1			
yes	409 (21.95%)	8 (50%)		3.56 (1.33‒9.53)		3.82 (1.42‒10.29)	

^a^Chi-squared test.

^b^Mann Whitney U test.

^c^Wald test.

### Follow up serological data

Three (i.e. No. 5, No. 11, and No. 14) out of 16 seropositive HCWs provided a second sample for serology testing. The time span between initial testing and the second sample was 36 (No.11), 57 (No. 14) and 85 (No. 5) days, which corresponds to 88, 118, and 128 days, post symptom onset (No. 5 and No. 14) or RT-PCR testing (No. 11). A decrease in SARS-CoV-2 IgG index value was observed in all three cases.

In addition, we analysed three initially seronegative HCWs, diagnosed with COVID-19 within a week following their first serology testing (i.e. in week 27). All three were epidemiologically linked and therefore considered to be clustered cases with intra-hospital transmission. These HCWs were immediately put on home isolation after being identified by RT-PCR testing (all experienced mild symptoms). After their return to work, testing for SARS-CoV-2 IgG was undertaken twice in convalescent plasma samples collected 34‒37 and 84‒85 days after the RT-PCR testing. A decrease in the index value was observed in all three cases between the second and the third month post symptom onset.

**Fig 2:**
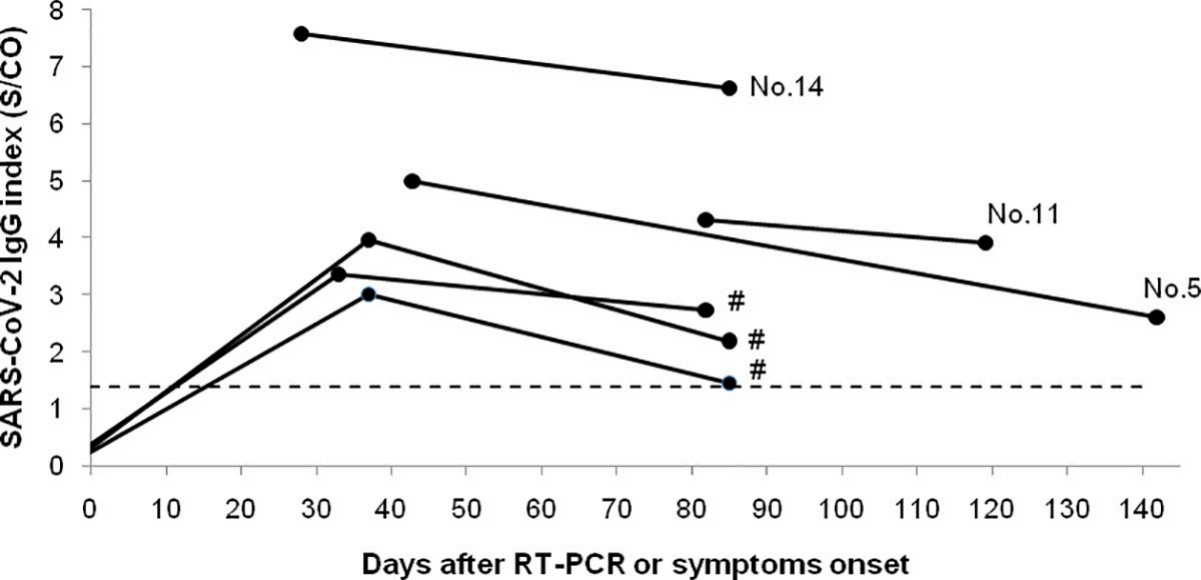
Follow-up serology testing. SARS-CoV-2 IgG follow-up data in 6 HCWs including three seropositive at initial testing (No. 5, No. 11, and No. 14) and three HCWs clustered cases (indicated by #), who had been confirmed with COVID-19 within a week following initial serology testing (in week 27). The dashed line indicates the positivity threshold (1.4 S/CO).

Overall, all six HCWs remained seropositive when the last test sample was collected at the median time of 86.5 days (range: 84‒128 days) post symptom onset or after the RT-PCR testing (Fig 2).

## Discussion

In Poland, a nationwide lockdown, including closure of schools and public institutions as well as mandatory wearing of a face mask, was implemented during the early stages of the COVID-19 pandemic [2]. As a result, the daily numbers of new SARS-CoV-2 infections were relatively stable up to the study period with a cumulative incidence of 90.6 per 100,000 as on July 1, 2020. Preceding the nationwide public health response measures, a set of infection prevention and control measures were implemented at the CMHI, to contain the spread of SARS-CoV-2 infection within the hospital. These included (but were not limited to): measurement of body temperature at hospital entry, patient/caretaker screening for SARS-CoV-2 RNA by RT-PCR on nasopharyngeal swabs on admission, and RT-PCR testing of all symptomatic staff coupled with contact tracing.

Seroprevalence studies in HCWs make it possible to assess the level of exposure, and indirectly, the effectiveness of the implemented protective measures. They are also crucial as an aid to health care resource planning to provide a safe environment to protect both patients and HCWs from SARS-CoV-2 infection. A few seroprevalence studies of HCWs in pediatric hospitals have been published so far. We present the results of serology testing in HCWs of the largest pediatric hospital in Poland, including over 82% of employees. In our study, the SARS-CoV-2 IgG positivity rate was 0.85%, and this was lower than in previous reports from pediatric hospitals. The seroprevalence of SARS-CoV-2 among 175 HCWs in a large referral pediatric hospital in Barcelona was 4% [10]. Another study form Italy, performed at the same time as the Barcelona study (i.e. mid-April 2020), showed a seroprevalence of 5.13% [12]. The timing of serology testing (April 2020 *vs* July 2020) and a significantly higher burden of COVID-19 (as Spain and Italy were two of the most affected countries in Europe [13]) may account for this difference. In addition, and in contrast to the Spanish and Italian reports, we did not have any confirmed COVID-19 cases among patients in our hospital until the end of the study period.

Interestingly, we did not observe any differences in seroprevalence between clinical and non-clinical working locations or across professional groups. This may be due to the fact that at that time the hospital did not manage any children with confirmed COVID-19. Although asymptomatic SARS-CoV-2 carriage among hospitalized children cannot be completely ruled out (as RT-PCR screening on admission is not 100% sensitive to preclude infection), the risk of children-to-staff transmission appears to be low. A recent study from a pediatric hospital in Chicago, revealed a low (1%‒2%) prevalence of SARS-CoV-2 among children without COVID-19 symptoms as well as no secondary transmission among HCWs exposed to those patients [14]. Another study, comparing the dynamics and determinants of SARS-CoV-2 transmissions among HCWs of adult and pediatric settings in Paris, revealed a significantly lower attack rate in a pediatric setting (2.3% *vs* 3.2%, respectively) [15].

In our study, prior RT-PCR testing, regardless of the result, was associated with an increased adjusted risk of SARS-CoV-2 IgG positivity. This may be a reflection of the fact that testing for SARS-CoV-2 RNA was performed as a part of contact tracing and in previously quarantined or isolated HCWs before returning to work. Considering that over 50% of seropositive HCWs in our study were asymptomatic or had no confirmed contact with a suspected or proven COVID-19 case, and that over 80% (13/16) had not been tested or tested negative for SARS-CoV-2 RNA, it could indicate that some SARS-CoV-2 infections among HCWs were unrecognized or undetected. Although false-positive SARS-CoV-2 IgG results are possible (e.g. due to crossreactivity to commonly circulating human coronaviruses) they are unlikely, even in the limited circulation of the virus [11]. Therefore, it appears to be reasonable to test both symptomatic and asymptomatic HCWs for SARS-CoV-2 RNA on a regular basis, if resources are available.

It is worth noting that the high percentage of asymptomatic cases among HCWs with evidence of SARS-CoV-2 infection observed in our study is in line with previous reports [10, 16].

In addition, we performed an exploratory analysis of antibody persistence in a subgroup of HCWs, who had been tested multiple times, to gain some insight into antibody persistence. SARS-CoV-2 IgG antibodies were detected up to 128 days post symptom onset or after the RT-PCR testing. However, the index value declined consistently in all subjects between the first and last plasma sample tested. Our findings are in line with some previously published data on kinetics of SARS-CoV-2 IgG [17, 18]. A recent study by Strömer et al. evaluated SARS-CoV-2 IgG levels in follow-up samples from 16 individuals (the median time of the last sample submission was 153 days after the RT-PCR testing) and revealed that several SARS-CoV-2 infected patients lost their N-specific IgG within a few months or could lose them soon [17]. It is worth noting that, in contrast to N-specific IgG, the response to the spike (S) protein was found to be more stable and was associated with the presence of virus-neutralizing antibodies (although at relatively low titres). Another study by Patel et al. evaluated the change in antibodies to SARS-CoV-2 over 60 days among 19 HCWs (including symptomatic ones), using an S-based assay [18]. They observed a decrease in anti-SARS-CoV-2 antibodies in all HCWs, with 58% of seropositive individuals becoming seronegative. Taken together, these findings suggest that seroprevalence studies may underestimate the rates of prior infections as antibodies may only be transiently detectable after infection. Nevertheless, the limited number of HCWs with follow-up sample available precludes any meaningful conclusion. In contrast to our study and the two studies mentioned above [17, 18], a recent population-based study, implementing two highly sensitive and specific assays to monitor antibody levels and their durability, indicated that antibody levels remained stable over four months. These discordant results may be attributed to sampling biases [19]. Therefore, larger longitudinal serological studies are necessary, including studies with virus neutralization assays, to explore the dynamics and the persistence of the SARS-CoV-2 antibodies as well as their correlation with immunoprotection from reinfection.

Our study has some limitations. First, testing was over a 5-week period (week 27‒32), potentially leading to changes in incidence over time and a possible variation in the professional groups attending the testing. Second, we cannot rule out that some HCWs were infected and either mounted no detectable antibody response or their antibody response had waned by the time of serology testing (we suspect this as we found one of the confirmed COVID-19 cases was seronegative 129 days after a positive RT-PCR test). Therefore, the seroprevalence in our study could be underestimated. Third, due to the lack of seroprevalence data for the general population, we cannot say whether the low seroprevalence observed in our study was attributed to the early implementation of infection control measurements at the CMHI or simply reflects the overall low SARS-CoV-2 seroprevalence in Poland. Fourth, the retrospective study design, prevented us from adequately adjusting for potential confounding variables and a cautious interpretation of the results is suggested. Moreover, the data on symptoms, exposure histories, and personal protective equipment use were collected only for the subset of seropositive HCWs by telephone interview as a part of the epidemiology investigation (subjected to recall bias), therefore more detailed information on risk factors could not be assessed. Nevertheless, to date, this is the largest study assessing the prevalence of SARS-CoV-2 IgG antibodies among HCWs in a pediatric hospital setting, with a high response rate and the use of a high-quality serological assay. Our study provides data on the seroprevalence of SARS-CoV-2 infection among HCWs in a pediatric setting during the initial peak of the pandemic, which provides information for control and prevention strategies for future waves of the COVID-19 pandemic.

## Conclusions

SARS-CoV-2 seroprevalence in healthcare workers of a tertiary pediatric hospital in Poland is low (0.85%). Further studies are necessary to elucidate the duration of anti-SARS-CoV-2 antibodies, as well as the correlation between seropositivity and protective immunity against reinfection. Regardless of the persistence of antibodies and their protective properties, such low prevalence indicates that this population is vulnerable to a second wave of the COVID-19 pandemic.

## References

[1] WHO Director-General’s opening remarks at the media briefing on COVID-19–11 March 2020. [cited 27 Oct 2020]. Available: https://www.who.int/dg/speeches/detail/who-director-general-s-opening-remarks-at-the-media-briefing-on-covid-19—11-march-2020.

[2] Week 42, 2020. [cited 26 Oct 2020]. Available: https://covid19-country-overviews.ecdc.europa.eu/#27_poland.

[3] WHO. WHO Coronavirus Disease (COVID-19) Dashboard | WHO Coronavirus Disease (COVID-19) Dashboard. In: Who [Internet]. 2020 [cited 27 Oct 2020]. Available: https://covid19.who.int/%0Ahttps://covid19.who.int/region/wpro/country/cn%0Ahttps://covid19.who.int/?gclid=CjwKCAjwztL2BRATEiwAvnALcpJvfJoB2ZO9AW4cscOjOPpuNNisqVVlTkpdslGJOuXSFkrhbLCafxoCjB0QAvD_BwE%0Ahttps://covid19.who.int/region/amro/country/br.

[4] Komunikat MPWIS z dnia 31 sierpnia 2020 r., przedstawiający sytuację epidemiologiczną na terenie województwa mazowieckiego związaną z koronawirusem SARS-CoV-2 wywołującym zachorowania na COVID-19.—WSSE. [cited 27 Oct 2020]. Available: http://wsse.waw.pl/aktualnosci-i-komunikaty/komunikaty/komunikat-mpwis-z-dnia-31-sierpnia-2020-r-przedstawiajacy-sytuacje-epidemiologiczna-na-terenie-wojewodztwa-mazowieckiego-zwiazan.

[5] Arevalo-Rodriguez I, Buitrago-Garcia D, Simancas-Racines D, Zambrano-Achig P, Del Campo R, Ciapponi A, et al. False-negative results of initial RT-PCR assays for COVID-19: A systematic review. Hozbor DF, editor. PLoS One. 2020;15: e0242958. 10.1371/journal.pone.0242958 33301459 PMC7728293

[6] Long QX, Tang XJ, Shi QL, Li Q, Deng HJ, Yuan J, et al. Clinical and immunological assessment of asymptomatic SARS-CoV-2 infections. Nat Med. 2020;26: 1200–1204. 10.1038/s41591-020-0965-6 32555424

[7] Ibarrondo FJ, Fulcher JA, Goodman-Meza D, Elliott J, Hofmann C, Hausner MA, et al. Rapid Decay of Anti–SARS-CoV-2 Antibodies in Persons with Mild Covid-19. N Engl J Med. 2020;383: 1085–1087. 10.1056/NEJMc2025179 32706954 PMC7397184

[8] COVID-19 in children and the role of school settings in COVID-19 transmission. [cited 26 Oct 2020]. Available: https://www.ecdc.europa.eu/en/publications-data/children-and-school-settings-covid-19-transmission.

[9] Amendola A, Tanzi E, Folgori L, Barcellini L, Bianchi S, Gori M, et al. Low seroprevalence of SARS-CoV-2 infection among healthcare workers of the largest children hospital in Milan during the pandemic wave. Infect Control Hosp Epidemiol. 2020; 1. 10.1017/ice.2020.401 32758311 PMC7438626

[10] Dacosta-Urbieta A, Rivero-Calle I, Pardo-Seco J, Redondo-Collazo L, Salas A, Gómez-Rial J, et al. Seroprevalence of SARS-CoV-2 Among Pediatric Healthcare Workers in Spain. Front Pediatr. 2020;8: 547. 10.3389/fped.2020.00547 33042908 PMC7516980

[11] Bryan A, Pepper G, Wener MH, Fink SL, Morishima C, Chaudhary A, et al. Performance characteristics of the abbott architect sars-cov-2 igg assay and seroprevalence in Boise, Idaho. J Clin Microbiol. 2020;58. 10.1128/JCM.00941-20 32381641 PMC7383515

[12] Amendola A, Tanzi E, Folgori L, Barcellini L, Bianchi S, Gori M, et al. Low seroprevalence of SARS-CoV-2 infection among healthcare workers of the largest children hospital in Milan during the pandemic wave. Infect Control Hosp Epidemiol. 2020; 1. 10.1017/ice.2020.401 32758311 PMC7438626

[13] Coronavirus Disease (COVID-19) Situation Reports. [cited 26 Oct 2020]. Available: https://www.who.int/emergencies/diseases/novel-coronavirus-2019/situation-reports.

[14] Patel AB, Clifford A, Creaden J, Kato K, Malakooti MR, Muller WJ, et al. Severe Acute Respiratory Syndrome Coronavirus 2 Point Prevalence Among Asymptomatic Hospitalized Children and Subsequent Healthcare Worker Evaluation. J Pediatric Infect Dis Soc. 2020 [cited 26 Oct 2020]. 10.1093/jpids/piaa102 32857134 PMC7499659

[15] Contejean A, Leporrier J, Canouï E, Alby-Laurent F, Lafont E, Beaudeau L, et al. Comparing Dynamics and Determinants of Severe Acute Respiratory Syndrome Coronavirus 2 Transmissions Among Healthcare Workers of Adult and Pediatric Settings in Central Paris. Clin Infect Dis. 2020 [cited 26 Oct 2020]. 10.1093/cid/ciaa977 33501952 PMC7454459

[16] Rivett L, Sridhar S, Sparkes D, Routledge M, Jones NK, Forrest S, et al. Screening of healthcare workers for SARS-CoV-2 highlights the role of asymptomatic carriage in COVID-19 transmission. Elife. 2020;9. 10.7554/eLife.58728 32392129 PMC7314537

[17] Strömer A, Rose R, Grobe O, Neumann F, Fickenscher H, Lorentz T, et al. Kinetics of Nucleo- and Spike Protein-Specific Immunoglobulin G and of Virus-Neutralizing Antibodies after SARS-CoV-2 Infection. Microorganisms. 2020;8: 1572. 10.3390/microorganisms8101572 33066057 PMC7650537

[18] Patel MM, Thornburg NJ, Stubblefield WB, Talbot HK, Coughlin MM, Feldstein LR, et al. Change in Antibodies to SARS-CoV-2 over 60 Days among Health Care Personnel in Nashville, Tennessee. JAMA—Journal of the American Medical Association. American Medical Association; 2020. 10.1001/jama.2020.18796 32940635 PMC7499233

[19] Alter G, Seder R. The Power of Antibody-Based Surveillance. N Engl J Med. 2020 [cited 26 Oct 2020]. 10.1056/NEJMe2028079 32871061 PMC7484745

